# Exposed necrotic bone in 183 patients with bisphosphonate-related osteonecrosis of the jaw: Associated clinical characteristics 

**DOI:** 10.4317/medoral.22133

**Published:** 2017-08-16

**Authors:** Leticia Bagan, Yolanda Jiménez, Manuel Leopoldo, Judith Murillo-Cortes, Jose Bagan

**Affiliations:** 1 Associate Professor of Oral Medicine. Universidad Europea de Valencia; 2 Professor of oral manifestations of systemic diseases. Valencia University; 3 Oral and Maxillofacial Surgeon. Consultant. University General Hospital; 4 University General Hospital, Valencia, Spain; 5 Professor of Oral Medicine, Valencia University. Head Service of Stomatology and Maxillofacial Surgery, University General Hospital. Fundación para la investigación del Hospital General Universitario de Valencia, Spain

## Abstract

**Background:**

The main objective of our study was to identify oral symptoms and signs most likely to be associated with the exposure of necrotic bone in bisphosphonate-related osteonecrosis of the jaw (BRONJ).

**Material and Methods:**

The study group consisted of 183 patients with BRONJ. We recorded data on the underlying disease, bisphosphonate used, location of osteonecrosis, symptoms, pain, fistula development, suppuration, infection, exposed necrotic bone, and BRONJ stage.

**Results:**

The mean age of the patients was 68.22 ± 12.19 years. The sample included 118 (64.5%) women. Breast cancer and multiple myeloma were the most common underlying diseases, and 50 patients received oral bisphosphonates for osteoporosis. Dental extractions (69.4%) and mandibular location (74.3%) predominated. The only two variables influencing the possibility of necrotic bone exposure were intravenous bisphosphonate administration and the presence of an intraoral fistula (*p* >0.05).

**Conclusions:**

Intravenous bisphosphonate use and intraoral fistula presence were associated with a major predisposition to bone exposure in patients with BRONJ.

** Key words:**Bisphosphonate, Osteonecrosis, Jaw.

## Introduction

In 2003, Marx (1) reported a series of 36 cases of osteonecrosis of the jaw following bisphosphonate treatment for cancer. Since then, many other authors have published case series and examined the etiological factors implicated in this severe oral complication, initially known as bisphosphonate-related osteonecrosis of the jaw (BRONJ) (2-8).

The scientific medical literature contains descriptions of risk factors for osteonecrosis (9,10). These risk factors include medication-dependent factors, the most important of which is the timing of bisphosphonate treatment. The concomitant use of other drugs, such as corticosteroids and antiangiogenic drugs, increases the risk of osteonecrosis (11). Local factors, such as tooth extraction (12-15), pre-existing periodontal problems, and implant or dental prosthesis use, can also predispose patients to BRONJ (9).

Clinically, exposed areas of necrotic bone may be observed; in other cases, only fistulas to the bone are detected. In stage 0 BRONJ, communication between the oral mucosa and the necrotic bone is absent (11,16-18).

Information on factors determining the presence of bone exposure is scarce. The main objective of our study was to identify the local oral symptoms and signs associated most frequently with the exposure of bone in BRONJ.

## Material and Methods

This retrospective study was performed at the Department of Stomatology and Maxillofacial Surgery, University General Hospital, Valencia, Spain, between September 2005 and December 2015. The study group consisted of 183 patients with BRONJ. The inclusion criteria were: (1) presence of at least one area of osteonecrosis on the mandible or maxilla and (2) diagnosis of BRONJ according to the criteria established by Ruggiero *et al.* (11).

We recorded data on the underlying disease, medications used, mode of bisphosphonate administration (oral or intravenous), and timing of medication use. We also collected data on patients’ symptoms, pain, presence of fistula, suppuration, infection, presence of exposed necrotic bone, and location of osteonecrosis on the mandible or maxilla. We determined the BRONJ stage in each case according to the criteria of Ruggiero *et al.* (11).

For the statistical analysis, we calculated means and percentages for the study variables. We performed a logistic regression anal-ysis to identify variables influencing the dependent variable, which was the presence or absence of bone exposure. We considered *p* < 0.05 to indicate statistical significance.

## Results

 Table 1 shows the clinical findings for the 183 patients. The sample comprised 118 (64.5%) women and 65 (35.5%) men with a mean age of 68.22 ± 12.19 years.

**Table 1:**
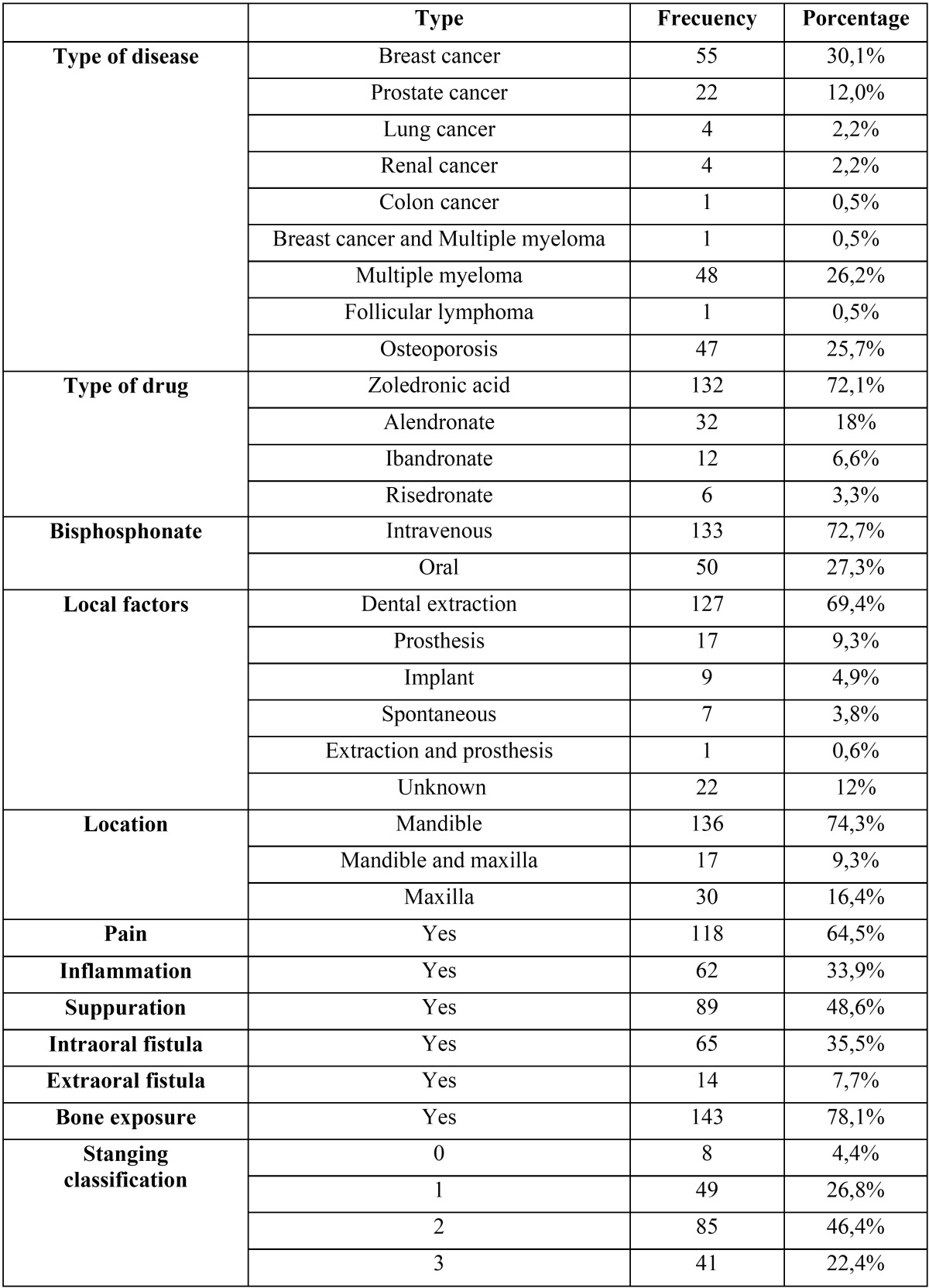
Clinical data for 183 cases of bisphosphonate-related osteonecrosis of the jaw.

The most frequent underlying diseases were breast cancer and multiple myeloma. Forty-seven patients were treated with oral bisphosphonates due to osteoporosis. Zoledronic acid was the most common treatment (72.1%). The mean duration of intravenous bisphosphonate therapy was 24.42 ± 19.27 months, and that of oral bisphosphonate therapy was 85.36 ± 54.97 months.

The most prevalent local predisposing factor for osteonecrosis development was dental extraction (69.4%), and mandibular loca-tion predominated (74.3% of sites).

Pain was the most frequently documented symptom (64.5%), followed by suppuration (48.6%), intraoral fistula (35.5%), inflammation (33.9%), and extraoral fistula (7.7%). Bone exposure was present in 78.1% of cases. Stage 2 was the most common stage of osteonecrosis (46.4%).

In the logistic regression analysis, necrotic bone exposure served as the dependent variable and age, sex, local factors, osteonecro-sis location, underlying disease, mode of bisphosphonate administration (intravenous or oral), and the symptoms and signs described above (pain, intraoral fistula, extraoral fistula) served as independent variables. The only two variables that influenced the possibility of necrotic bone exposure were the mode of bisphosphonate administration (intravenous or oral) and the presence of intraoral fistula (*p* < 0.05).

## Discussion

BRONJ is more common in women, due mainly to the large number of cases associated with treatment of breast cancer and osteoporosis (19). In our sample, 118 of 183 (64.5%) patients were female, and 55.3% of the sample had breast cancer or osteoporosis.

The mean age of patients with osteonecrosis is > 65 years (20,21). In agreement with this finding, the mean age of our patients was 68.22 ± 12.19 years (range: 32–89 years).

The risk of osteonecrosis is greater in oncologic patients receiving intravenous treatment than in those receiving oral bisphosphonates. Furthermore, the risk of osteonecrosis in patients with cancer treated with zoledronic acid is 50 to 100 times greater than that associated with placebo. The most common malignant diseases for which bisphosphonates are typically used are breast cancer, lung cancer, prostate cancer, and multiple myeloma (11). The most frequent disease in our series was breast cancer (30.1%), followed by multiple myeloma (26.20%). The percentages for other diseases, such as prostate cancer (12%), lung cancer (2.2%), and kidney cancer (2.2%), were much lower. Patients with osteoporosis comprised 25.7% of the sample, and all of the patients were women.

Most (72.1%) patients in our sample had been treated with zoledronic acid; 27.3% of patients had received oral bisphosphonates, such as alendronate (17,5%), ibandronate (6.6%), and risedronate (3.3%).

In our series, the duration of intravenous and oral bisphosphonate treatment was 25.42 ± 19.27 and 85.36 ± 54.97 months, respectively. The duration of oral bisphosphonate treatment greatly exceeded that recommended by experts.

The most common local predisposing factor for osteonecrosis is dental extraction, with 52–61% of patients reporting previous extraction due to periodontal or periapical infection (22,23). Our findings are in agreement with the majority of the published data; we found that dental extraction was a predisposing local factor in 69.4% of cases, followed by prosthesis use (9.3%), implant placement (4.9%), and spontaneous development or unknown cause (3.8%).

Osteonecrosis is known to occur more frequently in the mandible (74.3%) than in the maxilla (16.4%). However, both locations have been affected in a few reported cases (24). Most (74.3%) of our cases were mandibular, while 16.4% of patients presented with maxillary lesions, and 9.3% showed osteonecrosis in both locations. These percentages are similar to those reported by Kos *et al.* (24).

In 2007, Marx (25) reported a direct correlation between reported pain and clinical evidence of infection. In our series, 35.5% of patients reported no pain; these cases were classified as stage 1. The remaining 64.5% of patients reported pain, which was usually caused by associated infection (25).

In recent years, several modifications have been made to the osteonecrosis staging system. Ruggiero *et al.* (3) proposed one of the first classifications in 2006. In 2009, stage 0 was added to the classification, with the aim of including patients with nonspecific symptoms, such as pain, and those with other poorly defined clinical and radiographic alterations (26). In 2014, the classification was updated, with the addition of clinical features such as the presence of fistula to the bone in stages 1-3 (11). The presence of necrotic bone exposure is the main clinical feature in the most recent classification. We found this feature in 78.1% of our patients. In our series, 4.4% of patients had stage 0, 26.8% had stage 1, 46.4% had stage 2, and 22.4% had stage 3 disease.

The main objective of this study was to identify other clinical symptoms and signs influencing the presence of bone exposure in BRONJ using logistic regression. We found that the use of intravenous bisphosphonates and the presence of intraoral fistula were associated with a major predisposition to the development of bone exposure. From a clinical perspective, these features are the most significant of those examined in this study.
